# Detection of Potato Storage Disease via Gas Analysis: A Pilot Study Using Field Asymmetric Ion Mobility Spectrometry

**DOI:** 10.3390/s140915939

**Published:** 2014-08-28

**Authors:** Massimo Rutolo, James A. Covington, John Clarkson, Daciana Iliescu

**Affiliations:** 1 School of Engineering, University of Warwick, Coventry CV4 7AL, UK; E-Mails: J.A.Covington@warwick.ac.uk (J.A.C.); D.D.Iliescu@warwick.ac.uk (D.I.); 2 Warwick Crop Centre, School of Life Sciences, University of Warwick, Wellesbourne, Warwick CV35 9EF, UK; E-Mail: John.Clarkson@warwick.ac.uk

**Keywords:** FAIMS, soft rot, potato storage disease, early disease detection

## Abstract

Soft rot is a commonly occurring potato tuber disease that each year causes substantial losses to the food industry. Here, we explore the possibility of early detection of the disease via gas/vapor analysis, in a laboratory environment, using a recent technology known as FAIMS (Field Asymmetric Ion Mobility Spectrometry). In this work, tubers were inoculated with a bacterium causing the infection, *Pectobacterium carotovorum*, and stored within set environmental conditions in order to manage disease progression. They were compared with controls stored in the same conditions. Three different inoculation time courses were employed in order to obtain diseased potatoes showing clear signs of advanced infection (for standard detection) and diseased potatoes with no apparent evidence of infection (for early detection). A total of 156 samples were processed by PCA (Principal Component Analysis) and k-means clustering. Results show a clear discrimination between controls and diseased potatoes for all experiments with no difference among observations from standard and early detection. Further analysis was carried out by means of a statistical model based on LDA (Linear Discriminant Analysis) that showed a high classification accuracy of 92.1% on the test set, obtained via a LOOCV (leave-one out cross-validation).

## 1. Introduction

Crop wastage, through infection in vegetable stores, is a major issue and every year 5% of UK potato produce are disposed of because of storage bacterial infection [1]. One such infection is known as soft rot (bacterial soft rot is used for stored potatoes while blackleg for the growing crop). Bacterial soft rot can be caused by *Pectobacterium atrosepticum*, *Dickeya* spp. or *P. carotovorum* subsp. *carotovorum* [2–4], although recently it has been reported that *P. carotovorum* subsp. *brasiliensis* and *P. wasabiae* may also cause the disease in potatoes [5–7]. Monitoring the disease status of potatoes in stores is difficult, due to poor access. Current practice for detection of soft rot in store relies on visual inspection and smell. However, these stores are environmentally controlled, with air forced through the crop. Therefore, disease monitoring could potentially be achieved through the use of gas analysis with particular focus on volatile metabolites (also known as volatile organic compounds—VOCs). Previous work on early detection of potato storage diseases through gas analysis has been conducted over many years, dating back to the early work of Varns and Glynn in 1979 [8] followed by the study of Waterer and Pritchard from 1984 [9]. Most of this research has been based on GC (Gas Chromatograph) or GCMS (Gas Chromatograph Mass Spectrometer) in order to identify specific chemical compounds that might be characteristic markers of infected potato tubers [10–16]. However, a large number of different volatile biomarkers have been identified in many previous studies. These studies differ in hosts, pathogens, environmental factors, methodologies of experimentation, instruments limitations and usage, different analytical and data processing techniques, but above all, the relative time frames under consideration. In fact, according to Dixon *et al.* and Fiehn, plants produce about 200,000 volatile metabolites, both before and after harvest [17,18]. Wilson and Wisniewski claim that various environmental stresses can substantially increase the amount of volatiles that plants produce [19]. Moreover, GC and GCMS are expensive, require expert training, specialized carrier gases and therefore are unsuitable for continuous monitoring in real stores. We have tried to overcome the issues previously mentioned by employing a complementary approach, based on VOC fingerprinting rather than identification of individual VOCs. VOC fingerprinting in agriculture and forestry has been already investigated by means of electronic noses [20] and specific work was carried out in the past to study potato tuber infection with this approach [21].The work presented here reports the experimental results to assess the potential of FAIMS (Field Asymmetric Ion Mobility Spectrometry) technology for early detection of potato diseases in a laboratory setting. FAIMS and the electronic nose aim to identify patterns. FAIMS relies on a physical measurement of molecules (based on their mobilities in high electric fields), whilst the electronic nose uses a chemical interaction to produce response patterns. FAIMS technology is considered to be more sensitive, cost effective and also easier to use and deploy in a potato store compared with GCMS and traditional electronic nose. Most recently, in 2013, FAIMS instrumentation has also been used to detect citrus greening disease [22]. This is the first time FAIMS technology has been used to detect soft rot.

### FAIMS Technology

**Figure 1.**
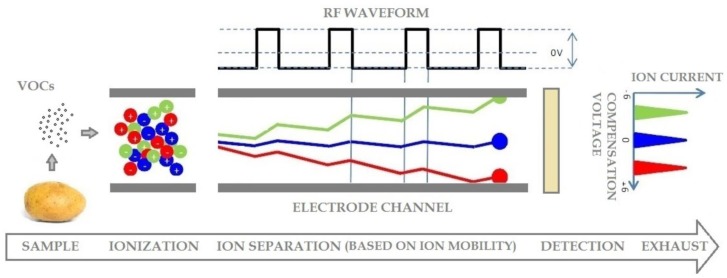
Basic working principle of Field Asymmetric Ion Mobility Spectrometry (FAIMS) [26].

**Figure 2.**
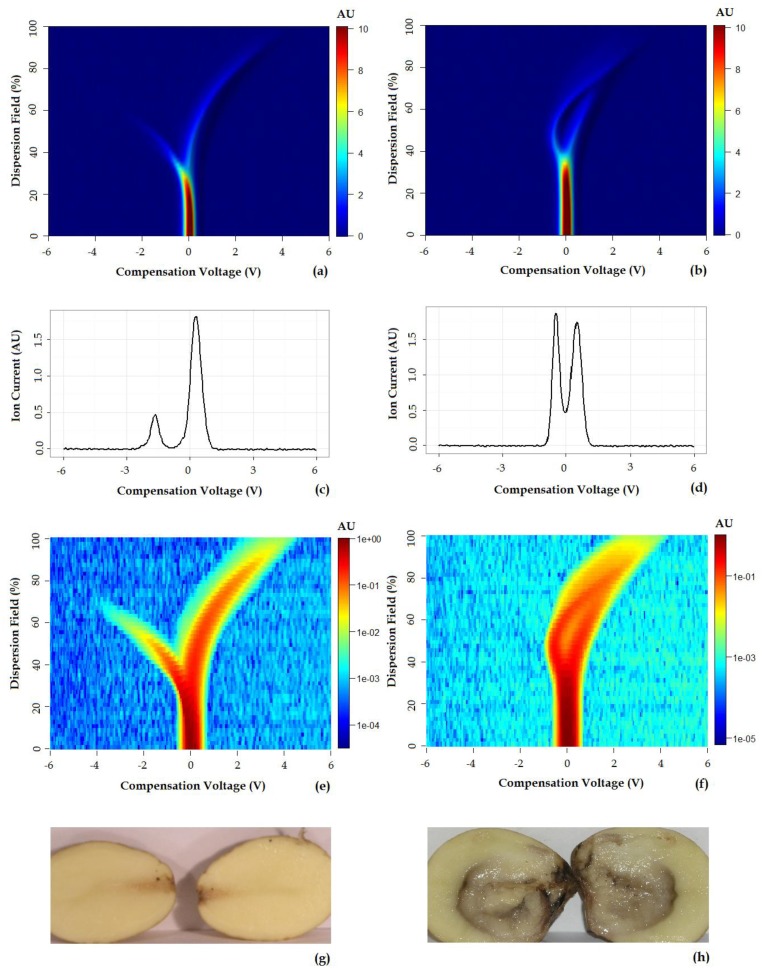
Control (**a**, **c**, **e**, and **g**) and tuber infected with soft rot (**b**, **d**, **f**, **h**). (**a**) and (**b**) are positive ion matrices while (**c**) and (**d**) show ion currents at 45% DF. (**e**) is the logarithmic representation on the ion current axis of (**a**), for control and (**f**) for the infected tuber in (**b**). Photographic analysis for control (**g**) and infected potato (**h**).

The purpose of an Ion Mobility Spectrometer is to separate complex chemical mixtures into individual or groups of chemicals based on their mobility in high electric fields. The basic working structure of an Ion Mobility Spectrometer (IMS) consists of three core parts, namely an ionization and reaction region (or ionization and reaction chamber), a drift region (or separation chamber) and a detection sensor. Once the sample molecules to be analyzed (in gas form) are ionized, they are pushed through the separation chamber to reach the detector [23]. The specific feature of FAIMS (Field Asymmetric Ion Mobility Spectrometer) is that an asymmetric RF (radio frequency) field is applied between two electrode plates through which the ions migrate (the RF field is orthogonal to the motion of the ions) in a saw-like trajectory towards the detector plate. Ions with the incorrect mobility touch one of the plates and lose their charge, while those with the right mobility collide with the sensor plate (with a less pronounced saw-like path, depicted in blue in Figure 1), generating an electrical signal, known as ion current [24]. Thus, due to the different mobility characteristic of each ion type, only a very restricted set of ions are able to exit the electrodes and reach the sensing plate. The utility of the instrument comes into play when a direct current field, known as the compensation voltage (or CV), is added to modify the asymmetric RF waveform in such a way to select different ions with specific ion mobilities (depicted in red and blue in Figure 1). Hence, by sweeping the CV, a mobility spectrum (ion current for the ordinate and compensation voltage for the abscissa, as indicated in Figure 2c,d) is generated for the chemical compounds under analysis. As some ions mobility is not constant with the applied electric field, the magnitude of the applied RF field (termed dispersion field or DF), is also modulated, thus a three dimensional spectrum is generated, (both compensation voltage and dispersion field are swept). By changing the polarity of the DF, the spectrum for positive or negative ions can be generated [25,26].

## 2. Materials and Methods

Potato tubers (variety Maris Piper) were inoculated with *Pectobacterium carotovorum*, in order to cause the pathology known as soft rot. Potatoes were first soaked in water for one hour before use and dried with a paper towel. Each tuber was stabbed at the stolon end with a sterile 200 μL pipette tip. To a 48 h culture of *P. carotovorum* grown on nutrient agar at 25 °C, 2 mL of sterile water was added and the colonies gently scraped (using a sterile plastic loop) to create a bacterial suspension. 20 μL (high inoculum) of this bacterial suspension was then pipetted into the stab wound in each tuber. A further set of tubers (controls) were stabbed at the stolon but not inoculated. After treatment the potato tubers were placed in sealed boxes at 25 ± 1 °C in an incubator (to allow rapid disease progression) and suspended on a mesh over water (400 mL), with the expected humidity to be above normal laboratory (neither absolute nor relative humidity levels were measured). No determination for latent *Pectbacterium* prior to infection was carried out, but controls were checked for infection throughout and at the end of the experimental procedure. The *Pectobacterium carotovorum* isolate used in this study was originally isolated by Dr. Glyn Harper (AHDB Potato Council, Sutton Bridge Crop Storage Research) and was isolated from an infected tuber, variety *Marfona*, and showing characteristic symptoms of bacterial soft rot. Isolated and in pure culture it caused pitting in CVP agar at 27 °C, was identified by PCR as P. *carotovorum* (*Pectobacterium* primer sets courtesy of Dr. J. Elphinstone, FERA, UK) and could infect potato tubers causing the original symptoms. No strain reference has been used to date for this isolate since it is the first time a paper has been published using it. The strain has been suggested by Dr Glyn Harper to be identified as SBEU_08.

### 2.1. Experimental Protocol: Sampling

For sampling, each tuber was placed into a PTFE (Polytetrafluoroethylene) container provided with a gas path inlet and outlet at either ends and sealed. Sampling was carried out for each individual tuber by allowing air to flow around it, with the mixture of air, gases and VOCs being fed for analysis to the FAIMS instrument (Lonestar, Owlstone Nanotech Ltd., Cambridge, UK) at a flow rate of 2 liters/min. Other FAIMS parameters included a dispersion field (DF) from 0 to 100% in 51 scanning steps and a compensation voltage (CV) from −6 to 6 V in 512 steps in order to build a 3D data matrix characteristic of the sample under analysis. Each potato tuber was scanned in such a manner twice. Prior to entering the sampling container the air was scrubbed clean and dried using moisture and hydrocarbon traps. PTFE containers were employed always separating usage for controls and infected tubers to avoid accidental cross contamination. The containers were also replaced, when appropriate, in order to eliminate potential byproduct of potato decomposition that might have affected results. After sampling each tuber was repositioned in the containing box in the incubator. Prior and after experimentation containers were thoroughly cleaned and sterilized.

After Preliminary trials used to establish an optimized time course, 4 sets of experiments were performed where 3 to 4 tubers were inoculated at days 1, 2 and 5 before testing, (with corresponding controls). The sampling procedure was carried out twice (two consecutive days). For the first experiment, on day one of testing, 18 potatoes were analyzed and the procedure was repeated on the second day with the same tubers, for a total of 36 samples. This procedure was carried out for all other experiments with 36 samples for the first three experiments, while the number was increased in the fourth one to 48 (24 tubers analyzed in the first day and again in the following day for a repeat). The number of potato tubers tested was 78 for a total number of samples of 156 (due to the repeats). Details of the experiments are shown in Table 1. The first part of the protocol had the objective to verify the ability of the Lonestar FAIMS to discriminate between controls and soft rot infected tubers sampled after 5 days of storage in the incubator, when the symptoms of the disease could be identified by olfactive, tactile and visual inspection of the sample. The second part of the experimental procedure had the aim to characterize early detection and consequently to probe the possibility of the instrument to detect the disease and discriminate between control and infection, when no visible, odor or tactile symptoms of the disease where present (one and two days post inoculation). Hence we used the terms “standard disease detection” (5 days post inoculation) and “early disease detection” (1 and 2 days post inoculation) respectively. At the end of the sampling procedure all tubers have been cut in half and photographed to gather indication of the degree of infection.

### 2.2. Data Analysis

With the parameters selected as indicated previously, for a single scan and for one type of ion matrix (either positive or negative) amounts to 51 × 512 data elements. For analysis, a single line of data points (one dimensional array) at 45% DF (dispersion field) was selected for each positive ions matrix thus yielding a number of variables of 512 for each sample. We have chosen the 45% DF since it accounts for most of the difference between controls and infected. Our decision to use a single line, instead of the whole data set, was because more lines, both for positive and negative ions, yielded no improved outcome when undertaking data analysis The second scan was used from each sample (two scans per sample were carried out), although the first scan could equally have been used.

For the analysis, two unsupervised techniques have been employed: PCA for dimensionality reduction and feature extraction while k-means for clustering. PCA (Principal Component Analysis) is a multivariate statistics technique in which (linear) data dimensionality reduction is carried out for a set of observations. A set of indices (known as principal components) represents a different linear combination of all the original variables. Each of the variables contributes with a different weight (or loading) to a principal component. The linear creation of each index is such that variability in the data is unevenly distributed among all indices (first principal components cater for the most variability) in such a manner to allow for few new variables (principal components) to represent most of the variation occurring in the larger set of variables, where the amount of variability retained depends on the linear combinations of variables and the whole data set [27].

The purpose of the k-means is to partition a N-dimensional population into k-sets and has been chosen instead of agglomerative hierarchical clustering because of its computational efficiency. After selection of the number of k groups, k initial seeds are randomly generated. After the selection of the k seeds, all samples are assigned to each seed according to the minimum Euclidean distance between the seed and each object. The whole process is iteratively repeated until optimal convergence is achieved [28]. In our case, we have used 2 initial (distinct) cluster centers for k. A 95% confidence interval was applied to the two centroids obtained with the k-means algorithm.

LDA (linear discriminant analysis) is a supervised statistical technique used as a classification model. The method relies on the calculation of the discriminant function for each category (or group) that the response variable can have [29]. A sample will be considered as belonging to one of the previously selected groups according to its discriminant score.. The analysis was performed using the statistical environment R (version 3.0.1, R Foundation for Statistical Computing, Vienna, Austria).

## 3. Results

**Figure 3.**
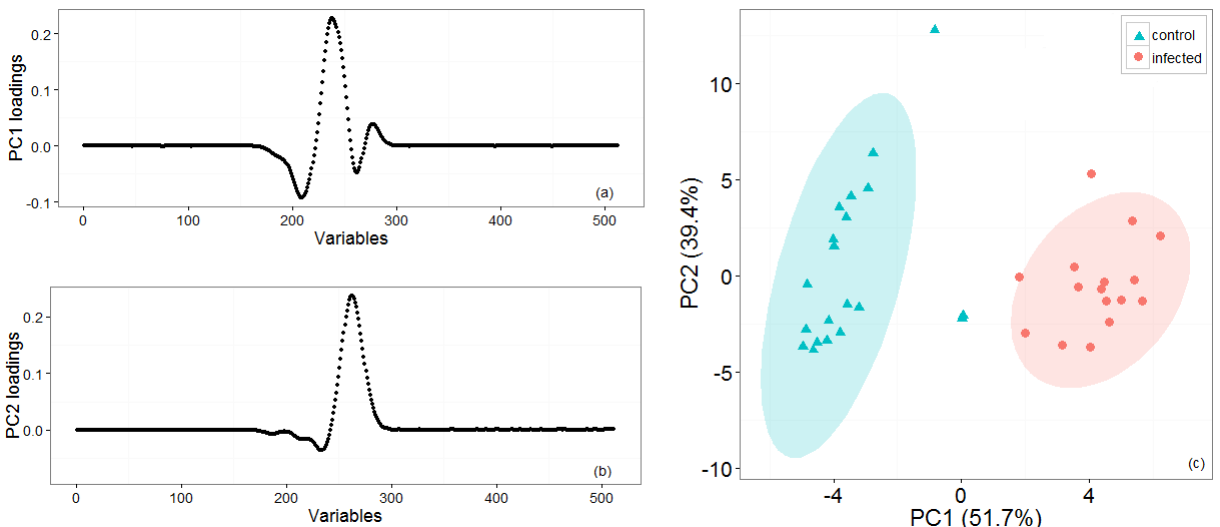
First set of experiments: Loading plots (**a**, **b**) for the two first principal components that account for most of the variance. (**c**) PCA and k-means clustering for two groups of potato tubers, with controls (cyan triangles) and infected (red circles) which have been grouped with 95% confidence ellipses around the centroid identified by the k-means algorithm.

**Figure 4.**
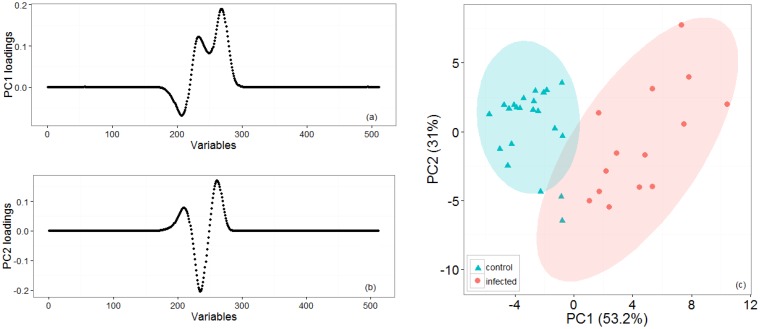
Second set of experiments: loading plots (**a**, **b**) for the two first principal components that account for most of the variance. (**c**) PCA and k-means clustering for two groups of potato tubers, with controls (cyan triangles) and infected (red circles) which have been grouped with 95% confidence ellipses around the centroid identified by the k-means algorithm.

**Figure 5.**
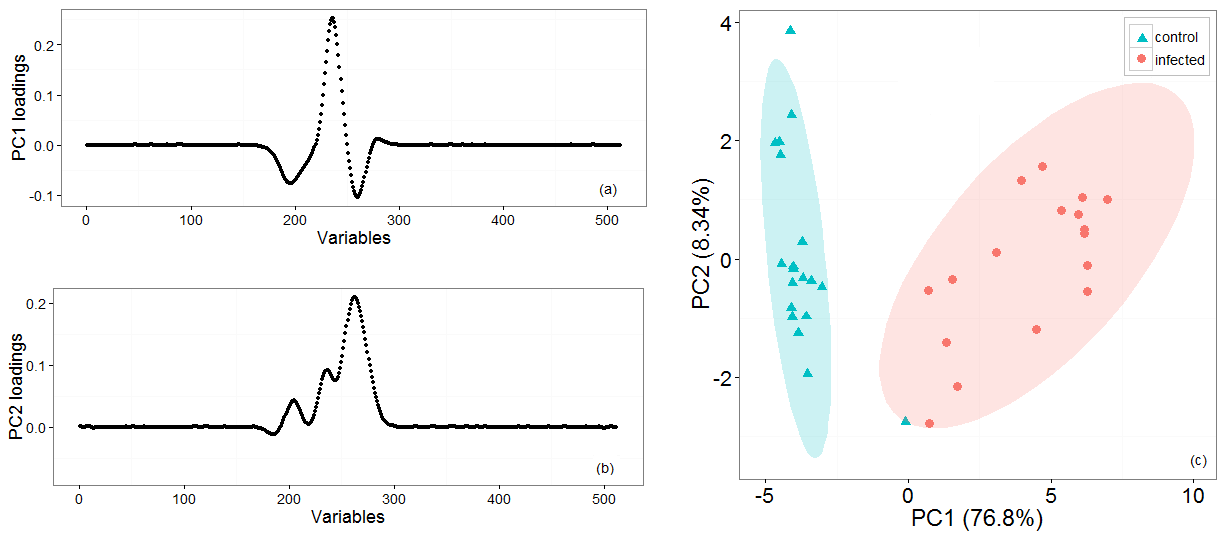
Third set of experiments: Loading plots (**a**, **b**) for the two first principal components that account for most of the variance. (**c**) PCA and k-means clustering for two groups of potato tubers with controls (cyan triangles) and infected (red circles) that have been grouped with 95% confidence ellipses around the centroid identified by the k-means algorithm.

**Figure 6.**
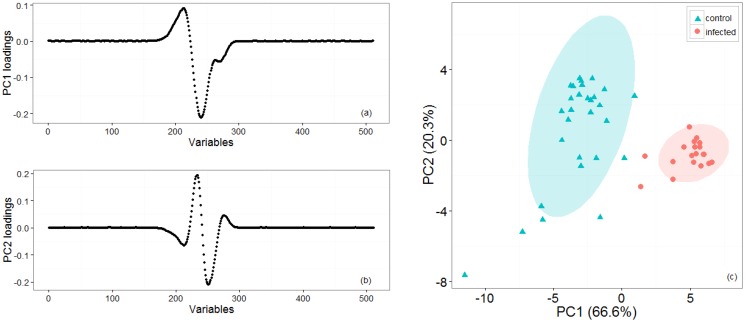
Fourth set of experiments: Loading plots (**a**, **b**) for the two first principal components that account for most of the variance. (**c**) PCA and k-means clustering for two groups of potato tubers with controls (cyan triangles) and infected (red circles) which have been grouped with 95% confidence ellipses around the centroid identified by the k-means algorithm.

**Figure 7.**
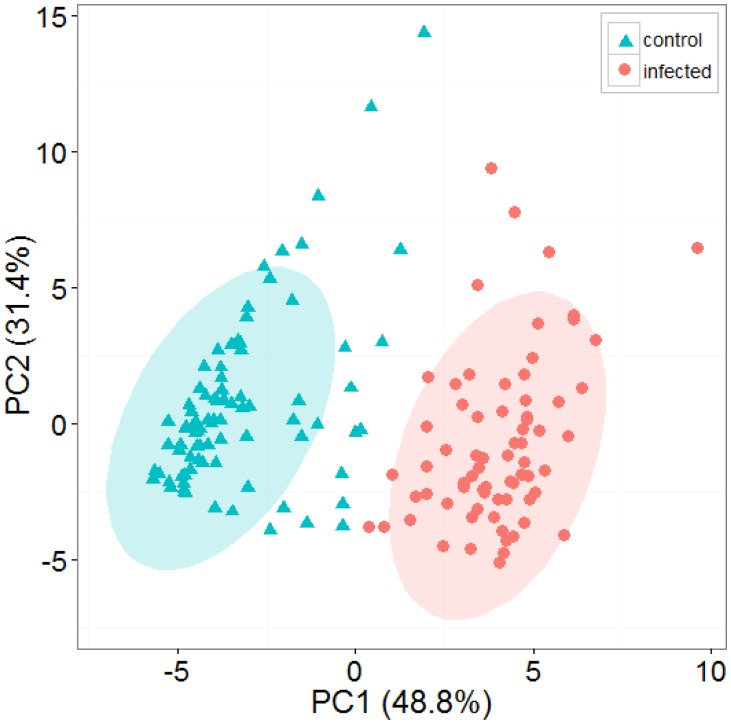
PCA and k-means clustering for the whole data set (two groups of potato tubers with controls (cyan triangles) and infected (red circles) that have been grouped with 95% confidence ellipses around the centroid identified by the k-means algorithm).

**Figure 8.**
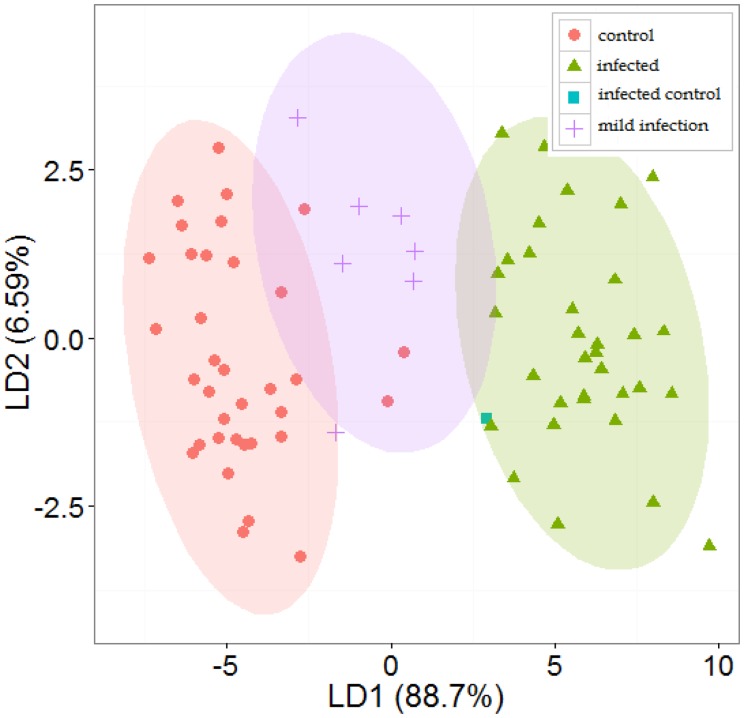
Predictive model based on LDA with 50% data as a testing set (there are three groups marked with a 95% confidence ellipse: control, mild infection and infection).

Figure 2 shows representative positive ion matrices (a, b), cross section for each ion matrix at 45% dispersion field (c, d) positive ion matrices (logarithmic base 10 for ion current axis) (e, f) and photographs (g, h), for a control tuber and an infected one as representative of two groups. Results for each of the four sets of experiments are shown in Figures 3, 4, 5, 6 and 7 while for the whole data set in Figure 7. In all graphs PCA and k-means clustering have been used for data representation. PCA scree plots for all experiments showed that most of the variance was explained by the first two principal components. Variance explained in the first two principal components is 91.1% and 84.2% for the first two experiments, 85.14% for the third and 86.9% in the last one. Results are presented in only two categories, “control” and “infected”, regardless of the time point when potato tubers were inoculated and sampled with the Lonestar. We have opted for this approach since no significant difference in instrument fingerprint could be identified for the different time points, thus showing the high level of detection capability of the Lonestar FAIMS for characteristic features of infection at 1 day post inoculation (sampling was carried out after 5, 2, and 1 days post inoculation) with our experimental conditions. Analysis of data was carried out by assuming no prior knowledge of the data. However, some controls showed clear signs of infection while a number of inoculated tubers manifested varying degrees of mild infection. Following a first analysis with PCA and k-means for the whole data set of the 4 experiments (Figure 7), we have tried to interpret the principal components by employing the Lonestar DF matrices and photographic analysis of the internal part of samples cut in half after the sampling procedure. By looking at the data sets we have noticed that the height of the Ion Current increases without any major shape difference going along the ordinate (*i.e.*, 2^nd^ principal component in Figure 7) while, instead of an intensity change, there a shape change was noticed along the abscissa. Hence, we believe that the first principal component appears to be correlated with a change in total volatile metabolites of the same type while the second principal component seems to be related to a change in the number of volatiles emitted. All samples outside the two confidences ellipses have shown intermediate characteristics between the two. Following this first analysis, we have proceeded to employ a classification model (based on LDA) that has been built considering three groups: controls, infected and mildly infected inoculated tubers (data points of infected controls have also been shown for clarity). The data have been split in half into training and testing sets (randomly chosen for each category) to build an LDA model. After training, the model has been evaluated with the test data and results are shown in Figure 8. Cross validation to estimate accuracy of the LDA has been carried out with a LOOCV (leave one out cross validation) repeated three times. Results have shown a classification accuracy of 90% for training and 92.1% for the testing set. LDA has been chosen over other techniques for the accuracy obtained and for being the only approach that does not require any model parameter tuning and optimization.

## 4. Discussion

Our results show that FAIMS technology is able to discriminate controls from tubers infected with *Pectobacterium carotovorum*, the most widespread pathogen affecting potatoes both in the field and in store. Identification of soft rot infection was achieved for samples 5 days post inoculation (“standard disease detection”) and after allowing for rapid disease progression, by storing potato tubers at 25 °C in a humid environment. Discrimination between infected tubers and controls was achieved also for samples 48 and 24 h post inoculation (“early disease detection”). The instrument yielded similar results in both cases, under the same experimental and data analysis conditions, thus indicating the potential of the technology not only for disease identification (at 5 days post inoculation) but also for early diagnostics (1 and 2 days post inoculation) for selected laboratory conditions. We wish to point out that our classification of results in two groups “standard” and “early” aimed to answer two core objectives of the work. The first one being if the technology could yield any result at all, and if so, how it could benchmarked with current practice of identification employed by farmers. The second aim was to identify how early this could happen when the other approach failed. We have found out that when no symptoms were identifiable by olfaction or visual inspection the instrument performed well and we opted for the definition of “early detection” thus yielding a more engineering based approach to the work. This does not necessary equate with a scientific biological analysis for early detection of the sample (with techniques such as PCR) nor our aim was to do so. However, the results were obtained with a specific and widespread variety of potato tuber (Maris Piper) and we realize that there are numerous varieties of potatoes that suffer by varying degrees to this particular infection. Further work will shed light on the FAIMS fingerprint that we have associated with soft rot as being caused either by volatile metabolites of generic breakdown products from the rotting tissue of a potato tuber or rather by specific metabolic activity caused by bacterial colonies inducing soft rot symptoms. Furthermore, we are also investigating if these chemical signals are common to this specific disease or common to a wide-range of bacterial infections. The whole data set was separated in two groups using both PCA and k-means analysis with 15% of samples outside the two 95% confidence intervals. The presence of these outliers can be attributed to different causes: varying biological conditions affecting each potato tuber differently, data processing, environmental conditions influencing the ionization of samples or the characteristics of the sensing unit. With regard to biology, a number of controls appeared infected while previously inoculated samples appeared to show varying degrees of disease progression. In the first case this was not unexpected as potatoes frequently carry soft rotting bacterial pathogens, such as *Pectobacterium*, on their surface. When the potatoes are wounded (as was done in the control treatments) and when they are put in a warm, humid environment, both these factors can result in soft rots occurring. We have estimated that 4% of controls showed to be highly infected, 5% intermediate characteristics of infection while of 10% of infected tubers showed only mild signs of infection, probably due to low bacterial colony growth. These observations were made by visual analysis of the symptoms of the tubers cut in half, standard practice among experts in the field and by analysis of size and distribution of the DF matrix of positive ions. No preliminary analysis on latent populations of bacteria was carried out. With regard to data processing, k-means is a computationally efficient algorithm, if compared to agglomerative clustering, but it is known to be sensitive to noise and outliers in the data set. In the data processing we have identified some of the outliers caused by the k-means algorithm. In addition, the PCA scree plots showed that most of the variance in the data set was accounted for in the first two principal components, thus indicating that the first two indices represent most of the variance for the whole data set. PCA loadings plots also indicated that most of the weight in the principal components can be attributed to a restricted number of variables estimated to be circa 30% (150 variables). However, further work needs to be carried out in order to assess the effect of environmental conditions on FAIMS instrument performance.

## 5. Conclusions/Outlook

Currently, there is no reliable non-destructive method for early identification of potato soft rot, either in store or in open field. Gas/VOC analysis may provide a possible solution. In this paper, we have reported for the first time the use of FAIMS gas analysis technology applied to this problem. FAIMS has the advantages of portability, high sensitivity and the ability to operate in harsher environmental conditions, such as those of commercial storage facilities. We have also explored successfully the combination of an unsupervised approach to mine patterns, interpretation of results and the development a robust but simple predictive statistical model with an accuracy of over 90%. Thus, FAIMS technology appears to hold promise for future use in early detection of potato storage diseases.

## Figures and Tables

**Table 1.** Experiments carried out and number of samples.

**Experiment**	**Number of Potato Tubers**						**Tests**	

	**1 Day Post Inoculation**		**2 Days Post Inoculation**		**5 Days Post Inoculation**			
			
	**Control**	**Infected**	**Control**	**Infected**	**Control**	**Infected**	**1^st^**	**2^nd^ (repeat)**
**No 1**	3	3	3	3	3	3	18	18
**No 2**	3	3	3	3	3	3	18	18
**No 3**	3	3	3	3	3	3	18	18
**No 4**	4	4	4	4	4	4	24	24
**Total**	13	13	13	13	13	13	78	78
